# Association between late-life hypercholesterolemia and progression of dementia severity among older adults

**DOI:** 10.1093/geroni/igaa057.3238

**Published:** 2020-12-16

**Authors:** Mo-kyung Sin, Yan Cheng, Ali Ahmed, Edward Zamrini

**Affiliations:** 1 Seattle University, Seattle, Washington, United States; 2 George Washington University, Washington, District of Columbia, United States

## Abstract

Progression of dementia severity varies widely by individuals and multiple factors might influence the progression. The aim of this study was to examine the relationship between late-life hypercholesterolemia and progression of dementia severity in older adults. We used prospectively collected longitudinal data from 2,686 adults aged ≥65 years in the National Alzheimer’s Coordinating Center. Progression of dementia severity was measured using both Clinical Dementia Rating (CDR) - Sum of Boxes (SOB) and Global scores. Kaplan Meier curves were plotted to estimate the association between hypercholesterolemia and progression of dementia severity. We also conducted multivariate Cox regression models to estimate the association of hypercholesterolemia with the outcomes adjusting for age, gender, race, ethnicity, marital status, living status, education, smoking, heart failure, atrial fibrillation, blood pressure, and diabetes. Hypercholesterolemia had significant association with CDR-SOB ≥ 1 point increase (unadjusted HR, 1.23; 95% CI, 1.13-1.35; p<0.001; adjusted HR, 1.17; 95% CI, 1.07-1.28; p<0.001). In addition, hypercholesterolemia had significant association with CDR-Global ≥ 0.5 point increase (unadjusted HR, 1.14; 95% CI, 1.04-1.25; p<0.001; adjusted HR, 1.11; 95% CI, 1.01-1.22 p=0.036). If these findings can be replicated in future studies, future studies need to examine if proper management of cholesterol may reduce the risk of Alzheimer’s dementia in late-life older adults.

